# Multiclass Image Classification Using GANs and CNN Based on Holes Drilled in Laminated Chipboard

**DOI:** 10.3390/s21238077

**Published:** 2021-12-02

**Authors:** Grzegorz Wieczorek, Marcin Chlebus, Janusz Gajda, Katarzyna Chyrowicz, Kamila Kontna, Michał Korycki, Albina Jegorowa, Michał Kruk

**Affiliations:** 1 Institute of Information Technology, Warsaw University of Life Sciences—SGGW, 02-787 Warsaw, Poland; michal_kruk@sggw.edu.pl; 2 Faculty of Economic Sciences, University of Warsaw, 00-927 Warsaw, Poland; jgajda@wne.uw.edu.pl; 3 Data Juice Lab sp. z o.o., 00-503 Warsaw, Poland; k.chyrowicz@datajuicelab.com (K.C.); k.kontna@datajuicelab.com (K.K.); m.korycki@datajuicelab.com (M.K.); 4 Institute of Wood Sciences and Furniture, Warsaw University of Life Sciences—SGGW, 02-787 Warsaw, Poland; albina_jegorowa@sggw.edu.pl

**Keywords:** multi-class classification, laminated chipboard, GAN, CNN

## Abstract

The multiclass prediction approach to the problem of recognizing the state of the drill by classifying images of drilled holes into three classes is presented. Expert judgement was made on the basis of the quality of the hole, by dividing the collected photographs into the classes: “very fine,” “acceptable,” and “unacceptable.” The aim of the research was to create a model capable of identifying different levels of quality of the holes, where the reduced quality would serve as a warning that the drill is about to wear down. This could reduce the damage caused by a blunt tool. To perform this task, real-world data were gathered, normalized, and scaled down, and additional instances were created with the use of data-augmentation techniques, a self-developed transformation, and with general adversarial networks. This approach also allowed us to achieve a slight rebalance of the dataset, by creating higher numbers of images belonging to the less-represented classes. The datasets generated were then fed into a series of convolutional neural networks, with different numbers of convolution layers used, modelled to carry out the multiclass prediction. The performance of the so-designed model was compared to predictions generated by Microsoft’s Custom Vision service, trained on the same data, which was treated as the benchmark. Several trained models obtained by adjusting the structure and hyperparameters of the model were able to provide better recognition of less-represented classes than the benchmark.

## 1. Introduction

The quality of a drill and its impact on the quality of a final product, which was a piece of furniture here, is of great importance in the production process. A drill that is not sharp enough should be replaced in order to prevent it from damaging the products, which would cause inconvenience and would generate costs to the producer.

The judgement of the state of a drill is not simple, and relying only on an expert’s eye would be quite risky. A traditional approach to this problem is collecting and measuring multiple signals produced by the drill, like the feed force, the cutting torque, the noise, the vibration, or the acoustic emission and then estimating its quality based on these signals [1]. This approach gives acceptably accurate results, as it was shown in previous works [2,3,4], but it requires the usage of multiple sensors. Many pre-processing operations have to be performed on collected data, such as calculating a number of statistical parameters of recorded signals or generating Fourier representations for specific feature selection [1].

In [5,6,7], it was shown that using only images of drilled holes and convolutional neural networks (CNN) can give satisfying results, and it is a much simpler solution than that based on multiple sensors. In [5], only an original set of 242 images was used. Trying to build simple CNN on dataset of this size achieved poor accuracy (35%) and required the use of highly pretrained neural networks on the model data in order to achieve a high value of said metric (around 93%). On the other hand, in [6,8], the data were expanded by simple image operations and data-augmentation techniques, such as converting an image to gray-scale values, adjusting the brightness of an image, adjusting its contrast, changing the hue, adding Gaussian noise or salt and pepper noise, creating reflections, or rotating and scaling an image. It was shown that expanding the dataset improved the results. In [7], the Siamese network was used to build the main classifier and to obtain the best classification results available.

Using only some types of transformations on existing data limits our final dataset to some extent. However, using data-generation techniques, such as generative adversarial networks (GAN), enables us to produce much more data. It was shown that the usage of GANs improves the results significantly [9,10].

In previous works, related to the data and the subject of our interest [5,6], a well-known AlexNet model was used to achieve accurate results. However, this model requires substantial memory resources, computing power, and training time. Recently we could observe an interesting trend of using relatively simple neural networks that can achieve similar, and sometimes even better, results for certain problems compared to more-complex networks, such as VGGNet, ResNet, and GoogleNet [11,12,13,14].

In the latest work on drill wear recognition [15], a set of models were compared to classify images into three classes. It was proved that due to unsatisfactory results for three classes of drill wear recognition, a division into two classes is sufficient from the business perspective, as misclassifying the worst class with the best class generates the greatest loss. However, a correct recognition of the middle class drill, which needs to be confirmed by an operator, can generate significant savings. It can still turn out to be of good quality, and a costly drill replacement may occur unnecessarily.

In this study, the usage of data-augmentation techniques and GANs was combined to produce more data, and a CNN model was proposed, to solve the problem of classifying drill images into three classes. What is more, unlike in the previous works, we did not use the *accuracy* classification quality measure to assess the quality of the model built on an imbalanced dataset, because this measure can erroneously reach high scores in the case of the presence of an overrepresented class in the dataset (the measure is correct only for that class) [16]. Instead, we compared models using measures better adopted to classification problems with under-represented groups. The measures introduced in [17], such as precision, recall, micro- and macro-area-under-the-ROC-curve (AUC) measures, F1 micro, and F1 macro were taken into account when comparing the resulting models. We also compared the results of the CNN model before and after adding more layers and collated it with the results achieved by the Microsoft Custom Vision model, which is much more complex than the CNN model. It was proved to be accurate in tasks of image classification [18], while being relatively simple to use and easily accessible, even to people unfamiliar with data science. In this study, we also proposed to use a correcting vector to adjust the prediction of classification quality for less-represented classes.

## 2. Methodology

In the following section, we discuss the methods used in the paper. Firstly, the methods used for data augmentation and the methodology of generative adversarial networks are recalled. Then, the convolutional neural network model is explained. In the next subsection, the Azure Custom Vision model is discussed. Finally, we present the testing framework applied to the models.

### 2.1. Data Augmentation

Data-augmentation techniques work well in image-analysis problems, improving the results [19,20], which was also proved to be true in the case of drill wear recognition [6]. In this study, we chose to use the following operations on images for data augmentation:

1. Colour to grayscale—in our dataset, colours were very similar to black and white and did not carry any relevant information that our model should learn. Since we chose to use CNN to classify images, we did not want our model to focus on learning colours. It was shown in [21] that using grayscale in CNN improves the results.
2. Horizontal and vertical flip—since our images were round holes placed in the centre of an image, we could use flips with no harm done to the quality of the picture [22,23].
3. Width and height shifted in the range of [−2,2]—a slight shift was used to make sure that the model did not learn to recognize the class based on hole placement. It is especially important when not every hole is placed in the very centre of the picture [22,23].

**Figure 1:**
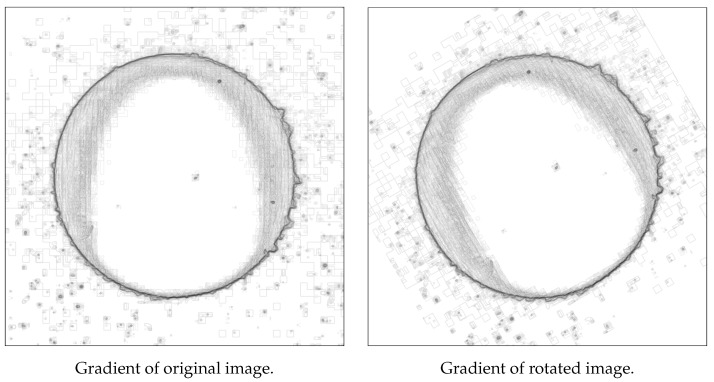
Comparison of gradients. Rotated image has visible blank spaces.

The rotation was omitted, due to the rectangular shape of all pictures. Revolution of an image erases the data in the corners. Introducing blank spaces will cause unwanted distortion in the dataset, as depicted in Figure 1.

Neither did we change the brightness or zoom, because we assumed that the pictures were taken in a specific lighting and that the distance between a camera and the chipboard with holes was set and stable. We did not want to artificially change a parameter that was actually constant.

The disadvantage of using only simple data-augmentation techniques presented above is that this can produce only a limited number of pictures. When all the combinations were used, the images began to duplicate. The number of pictures generated was not sufficient for applying GANs described in the next chapter. That is why, in order to boost our dataset (especially to increase the number of observations in the class of very fine holes, class 0), before applying data augmentation we made an additional transformation, which we proposed in this study.

The idea of the modification was to create additional observations by a combination of features from different existing images. New examples were generated by taking the linear combinations of corresponding points in a number of images.

This method was possible to apply only due to the fact that all examples used in the training of the model were photographs taken in reproducible conditions and had equal dimensions. What is more, it could be further explored by adding additional observations to every combination or by applying mixed weights to each of the contributing images, either predetermined or generated by random means.

### 2.2. Generative Adversarial Networks

Generative adversarial networks (GANs) were introduced by Goodfellow et al. in 2014 [24]. They proposed a model that consists of two parts, *G*—a generator—and *D*— a discriminator—which play a minimax two-player game. *G* learns to generate images possibly similar to original ones to maximize the loss of *D*, which in turn is learning to recognize the images it receives.

In order to create a fake example, which is represented by the generator’s distribution $p_{g}$ over data *x*, we had to define an input noise $p_{z}\left(z\right)$, mapping to the data space as $G(z;\;\theta_{g})$, where *G* is a differentiable function represented by a multilayer perceptron with parameters $\theta_{g}$. We can also define a second multilayer perceptron $D(x;\;\theta_{d})$, where D(x) is the probability that *x* came from the data rather than $p_{g}$. *D* is trained to maximize the probability of assigning the correct label to original examples and samples generated by *G*, while *G* is trained to minimize log(1−D(G(z))). So, *D* and *G* play a two-player minimax game with value function V(G,D):

(1) $$\min_{G}\max_{D}V\left(D,G\right)=E_{x∼p_{data}\left(x\right)}\left[\log D\left(x\right)\right)]+E_{z∼p_{z}\left(z\right)}\left[\log\left(1-D\left(G\left(z\right)\right)\right)\right]$$

The training of a GAN model is not a simple task to perform, due to a complicated nature of finding an optimal point upon which both *G* and *D* can converge on, which many studies describe as an equivalent to the search for Nash equilibrium in a high-dimensional, highly non-convex optimization space [25]. Both *G* and *D* need to be closely monitored and trained in relation to each other. If *G* were trained too much in relation to *D*, the results generated by *G* would be able to exploit specific local minima of *D*, converging all generated results to only a few examples with minimal variety, also known as a *mode collapse* [24]. With a too-exacting *D*, however, *G* will not be able to generate any results that would be able to pass the selection made by *D*, rendering all further training useless, due to aimless weight updates of *G*. This results in application of alternating both networks in alternate manner, by using gradient descent with respect to a cross-entropical loss function.

In this study, we trained three different GAN models, one for every existing class. This approach was chosen, even with multiple existing methods of generating multiclass images within a single GAN model, known generally as conditional generative adversarial nets (cGANs). CGANs, introduced by Mirza and Osindero [26], provide labels within the process of training, within the structures of generators and discriminators, in order to form a model that generates examples of a specific class, based on the provided noise input and class label. It was used to provide a method to multiply the training dataset to problems with a large number of available classes, without the need to train a network for every class separately.

This approach was rejected, since the potential gain attained by generating all artificial examples is not substantial, when the number of classes is relatively low, compared to other multiclass labelling problems (such as labelling the MNIST dataset [26]). Moreover, cGAN models are known to suffer from the tendency of overfitting the training data, in addition to mode collapsing and being biased towards the most-represented classes [26,27,28]. This approach also tends to have worse performance when compared to plain GAN models [26] and should be used as a method to save resources rather than an avenue towards generating better results.

### 2.3. Convolutional Neural Network Model

Convolutional neural networks are generally considered as the “go-to model” to apply to problems related to image processing, and they have proven to work well for image-classification tasks [29]. For the purpose of multi-class image classification, the CNN algorithm receives image data and produces a series of probability scores, corresponding to each label. Application of data augmentation along with additional examples provided by trained GAN models were able to fulfil the CNN’s requirement of having a broad dataset of examples to be trained on [30].

The CNN model acts as a derivative of the multilayer perceptron and is built with a series of convolutional blocks, which include convolutional layers, followed by activation functions and pooling layers.

Convolutional layers generate *n* feature maps by the application of linear combinations of several feature maps received as an input, where *n* is the number of filters in a layer. Filters of size $k_{w},k_{h}$ have their weights adapted in the process of training [31]. Application of filters of a size greater than 1×1 results in a reduction of size in the calculated feature maps. Additional hyperparameters of convolutional layers include the stride of the filters, the padding of the edges, and the choice of activation functions.

Subsequent convolutions of layers with a smaller size of the kernel are able to cover the same effective field of influence as a singular layer with a larger kernel size. Granulation allows the algorithm to determine more-complicated patterns with each passing layer, while reducing the number of parameters needed to train the model [32]. Pooling layers create a summary of p×p areas in feature maps created by previous convolutional layers. If the stride of $s\in N_{>1}$ is applied, the pooling layer reduces the spatial dimensions of feature maps generated by convolutions, greatly diminishing the amount of parameters needed for the CNN to train [31]. Pooling also allows the model to be indifferent to small changes in input maps and to gain transactional indifference, allowing for recognition of patterns that are not related to an exact placement where they appear [33].

The last block in the structure then connects to a number of dense, fully-connected layers of a neural network to provide the output [33].

### 2.4. Azure Custom Vision Model

The Microsoft Custom Vision service uses a machine learning algorithm to analyse images. Its functionality can be divided into two features, i.e., image classification and object detection. In the image classification, one or more labels is assigned to an image. In object detection, it is similar, but the coordinates in the image where the applied labels can be found are provided. In our case, we used the tool for image classification, and the results from this model were treated as a benchmark to compare with CNN models designed from scratch.

Implementing the Azure Custom Vision model is very simple and can be done by anyone, even by people unaccustomed to modern machine learning techniques. It can also be used in industry or other sectors to analyse image data, giving high quality results [18,34]. The prediction from the model has the best accuracy when the provided data are balanced. The task of the user is only to define class labels of the images when uploading the training dataset and to select the proper domain of the data. Exemplary domains are general, food, landmarks, retail, adult, and compact domains. The model is pretrained with data from the selected fields. Then, the model is evaluated on an independent test dataset. The results can be easily downloaded and explored.

Unfortunately, no further model description is available in Microsoft’s documentation of Custom Vision, apart from the fact that it is a CNN model. However, we cannot provide either the structure of the model or its hyperparameters.

### 2.5. Testing Framework

The original dataset was divided into the training set (60% of observations), the validation set (20%), and the testing set (20% of observations). The training dataset underwent the process of data augmentation, with the final training dataset consisting of:

- original images described in Section 3.1;
- images generated by the combinations of original images, described in the end of Section 2.1;
- images generated by traditional transformations described in Section 2.1 [22];
- images generated by GAN models described in Section 2.2.

Training of the CNN model was performed by the means of the *Keras* framework [35], with the loss metric set as a categorical cross-entropy loss (2), using the *adam* optimizer.

(2) categoricalcross-entropyloss=−∑i=1outputsizeyi·logy^i,

where

${\hat{y}}_{i}$—*i*-th prediction in the model output,
$y_{i}$—correct target value.

The performance of different combinations of hyperparameters were scored in two ways. Firstly, 10 models with the lowest categorical cross-entropy loss on the validation set were selected and compared. Secondly, as we dealt with the multiclassification problem and as we had a highly imbalanced dataset, we proposed an additional algorithm to select the 10 best models based on the ROC curves for validation data for each class. In the first step, we chose the minimum of the AUC values from all the three classes, and then we selected the models with the highest AUC values.

Then, confusion matrices for the best models were presented and the precision, recall, F1 score, and AUC measures were calculated for each class. Moreover, the accuracy (F1-micro score), the F1-macro score, the micro-average ROC curve, and the macro-average ROC curve were calculated to assess the overall performance of the model. These measures were presented with the validation data. The formulas are defined in (3)–(8) below.

(3) $$\mathrm{Accuracy}=\frac{TP+TN}{TP+TN+FP+FN}=\mathrm{micro}\;F1\;\mathrm{Score}\;,$$

(4) $$P\left(i\right)=\frac{TP_{i}}{TP_{i}+FP_{i}}\;,$$

(5) $$TPR\left(i\right)=R\left(i\right)=\frac{TP_{i}}{TP_{i}+FN_{i}}\;,$$

(6) $$FPR\left(i\right)=\frac{FP_{i}}{FP_{i}+TN_{i}}\;,$$

(7) $$F1\left(i\right)=\frac{2\times P(i)\times R(i)}{P(i)+R(i)}\;,$$

(8) $$mF1=\frac{\sum_{i=1}^{n}F1\left(i\right)}{n}\;,$$

where

TP—the number of true positives.
TN—the number of true negatives.
FP—the number of false positives.
FN—the number of false negatives.
$TP_{i}$—the number of true positives for class *i*.
$TN_{i}$—the number of true negatives for class *i*.
$FN_{i}$—the number of false positives for class *i*.
$FN_{i}$—the number of false negatives for class *i*,.
P(i)—the precision for class *i*.
R(i)—the recall for class *i*.
TPR(i)—the true-positive rate for class *i*.
FPR(i)—the false-positive rate for class *i*.
F1(i)—the F-1 score for class *i*.
mF1—the macro F-1 score.
*n*—the number of classes.

The AUC measure was determined by plotting the true-positive rate (5) against the false-positive rate (6) from confusion matrices generated at various threshold values. In this study, five different ROC curves were generated for every model. Three ROC curves were generated by examining predictions generated for every existing class. The micro-average values of the true-positive rate and the false-positive rate were calculated by concatenating all predictions and scores into a binary classification problem, thus generating a ROC curve that displays weighted average of previously mentioned ROC curves. The macro-average ROC curve displays the mathematical average of ROC curve graphs generated for every class and was generated by averaging all true-positive rates for every present point on the false-positive rate axis [36].

The output of the model for each image is a vector of probabilities $p_{i}=\left[p_{0},\;p_{1},\;p_{2}\right]$, where $p_{0}$, $p_{1}$, $p_{2}$ is the probability that the observation *i* belongs to class 0, class 1, and class 2, respectively. The default assignment of an observation to a class is the class with the highest probability ($max\{p_{i}\}$).

As our dataset was imbalanced between classes, the appropriate method of reducing the bias happening within the CNN network is introduced. An ensemble of methods known as threshold moving or post-scaling involves adjusting the probabilities retrieved from the output of the model by an artificial measure, usually based on prior class probabilities [37,38]. In this study, the correcting vector was defined as π:

(9) $$\begin{array}{cc} \pi & =[\pi_{0},\;\pi_{1},\;\pi_{2}]\;,\\ \pi_{n} & =\frac{1}{|c_{n}|}\;, \end{array}$$

where $|c_{n}|$—size (number of objects) of class *n*.

The probabilities after the correction was done took values resulting from Equation (10). The final allocation again assigns the class with the highest probability to the observation.

(10) $$p_{i}^{\mathrm{corr}}=p_{i}^{\mathrm{orig}}\times \pi ,$$

where

$p_{i}^{\mathrm{corr}}$—vector of probabilities after correction,
$p_{i}^{\mathrm{orig}}$—vector of original probabilities,
π—correcting vector.

As we changed the proportion of observations in each class in the training set, we applied the correcting vector in two ways and selected the one with the more-rewarding results. Firstly, we took the size of the original dataset with the created combined images (class 0: 19; class 1: 162; and class 2: 93) and then the size from the fully augmented training dataset (class 0: 3201; class 1: 7887; and class 2: 4601).

The final model selection was made by re-comparison of all the measures for the three models after application of correcting vectors, and the best performing solution was benchmarked with the solution proposed by the Microsoft Custom Vision tool. As we did not want to favour any solution, the correcting vector was also applied for the Custom Vision model. The selection of the vector was made based on the independent validation set.

## 3. Experiment

Within this section, the conducted experiment is described. Firstly, a description of the original dataset is provided. Secondly, the results of data augmentation and data generation with GANs are presented. What is more, we discuss the outcomes from the Azure Custom Model and the CNN model built from scratch.

### 3.1. Data Description

The initial dataset consisted of 459 images of drilled holes for three classes. The data were collected in cooperation with the Faculty of Wood Technology of the Warsaw University of Life Sciences—SGGW, using a standard Buselatto JET 100 CNC vertical machining centre. Holes were drilled in a standard laminated chipboard (KronopolU 511 SM) with dimensions 150×35×18 mm by using a regular 12 mm FABA drill equipped with a tungsten carbide tip.

Appointed experts assessed the state of every photograph taken at the workstation, based on the condition of each hole, thus dividing the dataset into three classes:

- Class 0—finest quality among the classes; it showed little or no damage around the edges.
- Class 1—few instances of bigger fuzz/ripping present; the quality of the hole was still acceptable but should be confirmed by an operator.
- Class 2—larger damage around the perimeter of the hole; larger cases of torn material; unacceptable condition; drill should be replaced.

**Figure 2:**
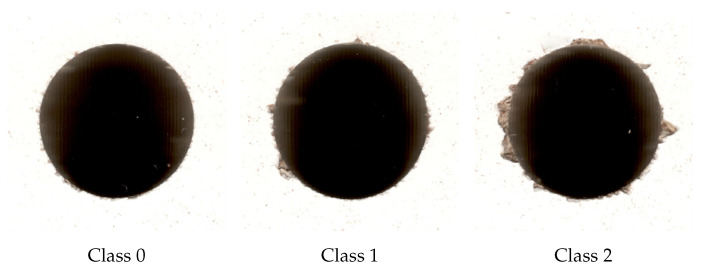
Exemplary images from original dataset.

Example images from class 0, 1, and 2 are shown in Figure 2. Sizes of classes in the original dataset are shown in Table 1.

We divided the original dataset to the training, validation, and testing sets as shown in Table 2. The validation dataset was created to choose the optimal structure of CNN, and the test dataset was used to assess the selected models on independent observations. The number of observations in the validation and test sets were pretty low, but we wanted to keep original images in these samples, as we believed that the evaluation of the model should be done on real data. Diminishing the number of original observations in the training set would also be a harm to the diversity of new images created using data-augmentation techniques and GANs and, as a result, could reduce the performance of the models.

**Figure 3:**
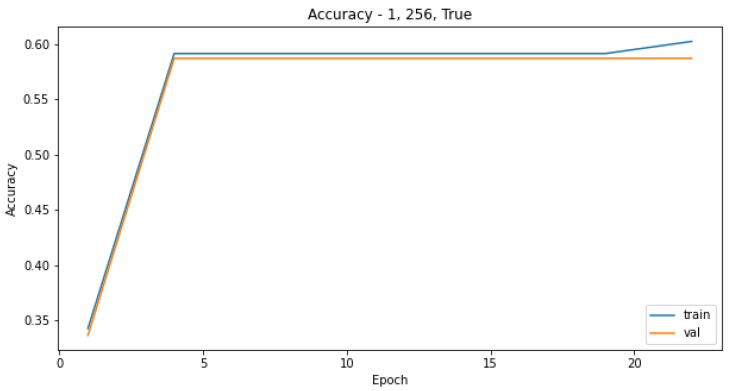
Model accuracy for the training and validation sets in relation to different number of epochs used to train the CNN.

**Figure 4:**
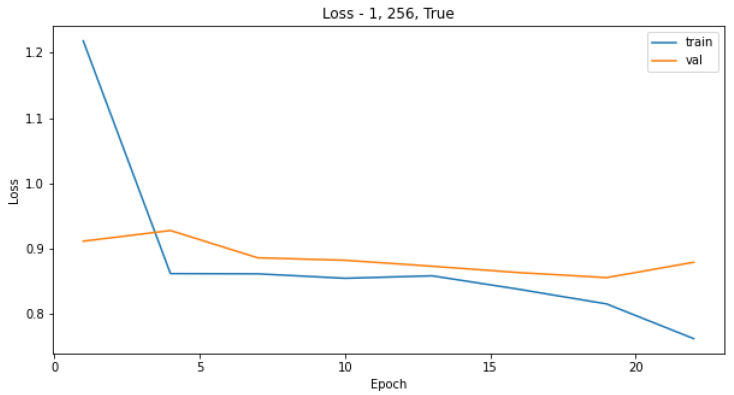
Model loss for the training and validation sets in relation to different number of epochs used to train the CNN.

Attempts to create a CNN model only on the original training dataset were not successful, as the most common result was for the model to converge on only predictions of the most-represented class as seen in the confusion matrix in Table 3. Increasing the number of epochs did not influence the performance of the model, as it reached the plateau quickly, and the predictions for the validation set were the same for each epoch, as seen in Figure 3 and Figure 4. The reason for that is probably the highly imbalanced dataset.

The training dataset was enlarged with the transformations described in Section 2.1. As the images of the drilled holes were not very complicated and detailed, we decided to reshape them to the size of 80×80, which made the computation less expensive and faster, especially in the case of training the GANs and the CNN model. We found out that GANs are better in producing lower-resolution images, and we concluded that 80×80 is an optimal resolution for our dataset. It is low enough for a GAN to train and to achieve satisfying results but high enough to capture the features specific for each class and to distinguish well between them. In the following subsections, the results of the application of the data-augmentation and the GANs techniques is presented.

### 3.2. Data Augmentation

Before we trained GANs to produce more data, we wanted to broaden our initial dataset by combining images using the described transformation and by performing a few simple data-augmentation operations on it. For class 0, due to a small number of observations in the original dataset, the data were multiplied by combining images with the application of different sets of weights for both components, as described in Section 2.1. The usually used weight of (1/2,1/2) will create images equally different from both pictures and is a better solution than applying for example (1/4,3/4), where the transformation favours the picture with a larger weight equal to 3/4. However, class 0 has only 19 observations, and applying equal weights did not produce enough observations; so, for this class, we used two sets of weights: (1/2,1/2) and (1/4,3/4). Then, the data augmentation was applied.

**Figure 5:**
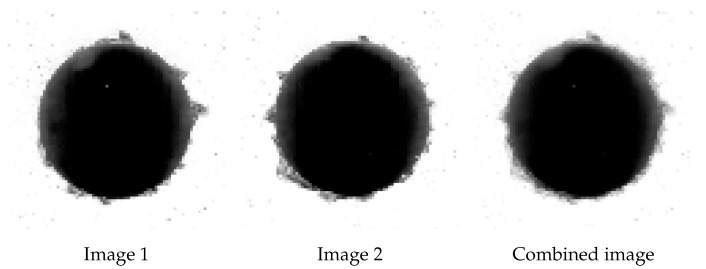
Creation of additional data entry for images belonging to class 2.

Classes 1 and 2 were multiplied only once with the weights equal to (1/2,1/2), and, again, data-augmentation techniques described in Section 2.1 were applied. An example of an image generated by this method is presented in Figure 5.

The operation of combining halves of images produced many new observations if the original dataset was numerous (e.g., for class 1 with 162 original images we obtained 13,041 new pictures), so we randomly chose 11% of images created for class 1 and 10% for class 2. The percentage of images selected was dependent on the result of data augmentation. If the number of duplicated images was too high, it was necessary to increase the number of data produced by the transformation. The dataset consisting of original images with additional instances created by this transformation is described in Table 4.

**Figure 6:**
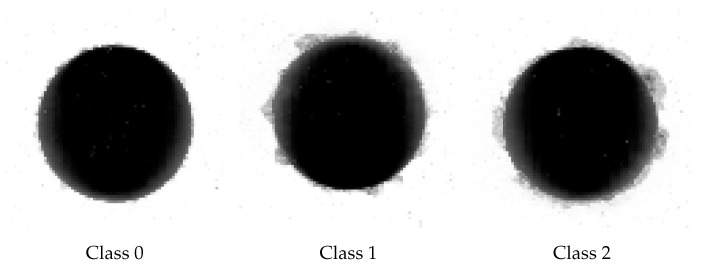
Images generated using simple data augmentation techniques.

Since the process of data augmentation, described in Section 2.1, is performed using randomly chosen sets of limited number of transformations on randomly selected images, created dataset can seldom produce duplicated images. We filtered unique observations to be assigned to final training set. The parameters described before were set to obtain the multiplication of observations in class 0 and 2 four times and in class 1 two times, compared to original counts of images. A decision on the number of multiplied images was made after applying many tests which aimed to find the optimal number of observations required for training GANs. Trade-off between data originality and minimum number of observations needed to train GANs had to be found. As a result, in the final training dataset we reduced the effect of imbalanced dataset. Finally, we got a train dataset consisting of the number of observations described in Table 5. Exemplary images generated using these operations are shown in Figure 6.

### 3.3. Data Generation Using GANs

We decided to use GANs to expand our initial dataset further, as it can bring more diversity to the training data than simple transformations, and the generated images should be similar to the original ones. We constructed the generator and the discriminator model having the structures shown in Table 6, Table 7 and Table 8.

#### 3.3.1. Generator

The structure of the generator takes any number of images of size 80×80 as an input. This data gets transformed into a fully connected layer that gets reshaped into 128 examples of downscaled-to-5×5 images that were generated as a base for the output image. Each channel then gets upscaled in both dimensions within the process of deconvolution. After achieving proper dimensions, the final convolution layer transforms data from all channels into a variable number of generated images of size 80×80. It has to be noted here that the model described in Table 6 was not compiled and trained on its own, as the output of this model was fed directly into the discriminator, as described in Table 8.

#### 3.3.2. Discriminator

The discriminator serves its purpose as the expert deciding whether the generated image comes from a real dataset or was generated artificially, by feeding the data through a convolutional neural network, as described in Section 2.3. The structure of the network from Table 7 shows that the discriminator takes the generated image of size 80×80 from the generator; filters it twice through two convolutional layers with the stride equal to 2, thus reducing their dimension size by the factor of 2 with every convolution; and then decides in a single fully connected layer whether the image is real or fake.

#### 3.3.3. Final Model

In the final model, described in Table 8, it has to be noted that only the weights belonging to the generator model were trained for this specific model configuration. As described in Section 2.2, the discriminator had to be trained beforehand on a manufactured collection of real examples mixed with outputs of the GAN model from the previous epoch. Here, the next iteration of the generator model was adjusted, in order to minimize losses by generating images that passed as *real* to the previously trained discriminator. The final model was compiled with an *Adam* optimizer (learning rate =0.0002 and beta =0.5) and with the binary cross-entropy loss function.

Three GANs were trained separately, one for each class. They were trained on the training set described in Table 5. Each GAN had the same structure and hyperparameters described above, but they differed in batch size and the number of epochs. For each class, the best model was chosen based on the values of discriminator and generator loss and expert’s opinion on the quality of images produced. The goal was to produce images possibly similar to original ones, having features characteristic for each class and possibly different between classes.

**Figure 7:**
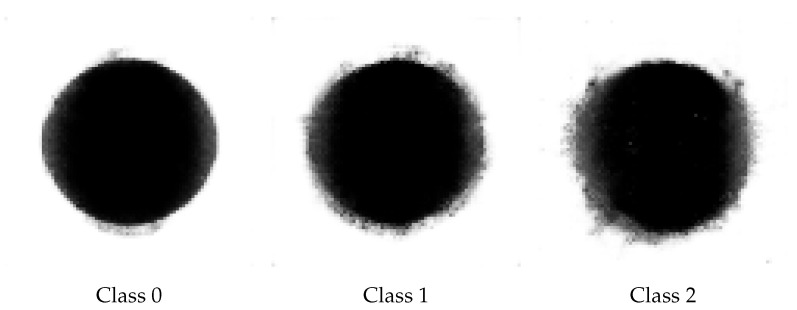
Images generated by the GANs.

The first GAN was trained for class 0 on 1440 images from the broadened train set. The optimal batch size for this model occurred to be 16, and the best results were achieved after 40 epochs of training. Discriminator loss turned out to be 0.797 and generator loss 0.607; the values were close to each other, which means that neither model dominated the other one. The Second GAN was trained for class 1 on 3190 images from the broadened train set. The optimal batch size for this model occurred also to be 16, and the best results were achieved after eight epochs of training with a discriminator loss of 0.720 and a generator loss of 0.722. The values are even closer to each other than in the case of class 0, which may come from the fact that GAN was trained on a larger dataset. The third GAN was trained for class 2 on 2080 images from the broadened training set. The batch size chosen for this model was again 16, and the number of epochs was 65. The loss value for the discriminator was 0.684 and for the generator was 0.750, which again is quite close to one another. Exemplary images produced by the trained GANs are shown in Figure 7.

We can see that the generated pictures definitely differed from each other between classes, and the characteristic features for every class were preserved. The edge in the first class is clearly the smoothest as in the original data, and it is getting progressively worse for classes 1 and 2. It is worth noticing that, for class 2, the generated images differed significantly from the original ones, and this can be a problem in the process of training the final model. Exaggerated features generated by the GAN network can skew the model into the state where real data will be assigned to the milder class. This was tested within the analysis of the model responses on the validation and testing datasets.

The distribution of data generated by the GANs is shown in Table 9. The number of observations in each class was set to be similar to the number of images after data augmentation. We did not want to create too many pictures from classes with a small amount of original observations, as it would cause the danger of creating many images that do not differ in any way from each other. After many tests, the selected proportions were treated as optimal.

#### 3.3.4. Final Training Dataset

We slightly changed the distribution of data between classes in comparison to the proportions of the dataset consisting only of original images (Table 1). In this situation, it has to be noted that generating many images from a small number of pictures in a less-represented class can create small variance in the resulting pictures, while generating images in a better-represented class can increase the variance in images in this class. On the other hand, boosting the number of observations in a less-represented class may help the model to better recognize the class. The final distribution of data in each class and the dataset is presented in Table 10.

The number of observations used in modelling may be questionable, as the dataset was still not fully balanced. We could have produced more images for classes 0 and 2, but, as mentioned before, we did not want to affect the variance between the observations. It may also result in overfitting the model. Moreover, by keeping class 1 as the most-numerous one and class 0 as the least-numerous one, we taught the model to be sensitive to the disproportion in data, which also occurs in the validation and test sets and, what is more important, in real data, if the model is be used in production in the future.

### 3.4. Azure Custom Vision Model

In order to set up a hard benchmark, we used Microsoft’s Custom Vision service for image classification and trained it on our final training dataset. We are aware of the fact that the model should be trained on the balanced dataset, but it was impossible in the case of our data, which was explained in previous sections. Moreover, real data are rarely balanced, so the model would be tested on more real business environments. We applied the correcting vector to the predictions to reduce the impact of this drawback. The following results are shown on the validation dataset, on the basis of which we would choose the best version of the model. This provided the possibility to compare the final CNN model with the Azure model on an independent dataset for both solutions.

**Figure 8:**
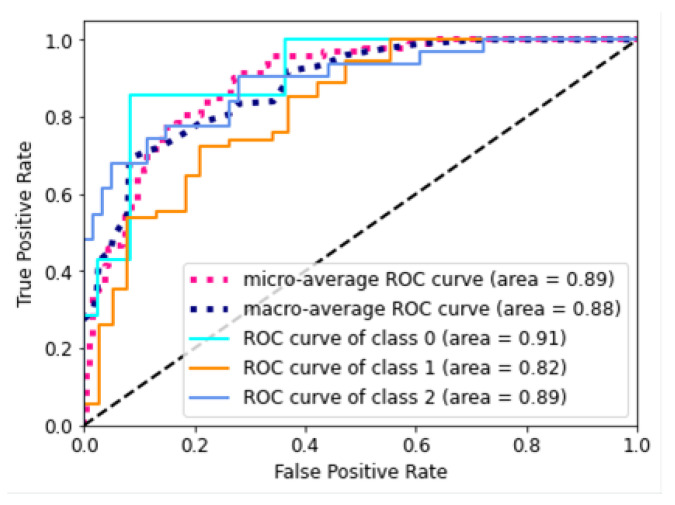
ROC curves for the Microsoft Custom Vision model.

The ROC curves presented in Figure 8 suggest that the model is of a good quality, and it was able to predict all the classes. Both micro- and macro-average ROC curves are located high in the coordinate systems, which means that the model is not focused on predicting only the observations from the largest group.

From the confusion matrices presented in Table 11, we can see that, on the validation set, the model with default predictions favoured class 1. This is understandable, since the dataset was imbalanced and class 1 was overrepresented. After applying the correcting vector, the results were slightly more satisfying; the model could predict more images from classes 0 and 2. The model with original predictions tended to generalize its responses towards marking ambiguous examples with the most-common class. This is not a desired behaviour, which is why the corrected models are considered as superior when compared to the default results, even if the predictions that deviate from class 1 are not entirely correct.

The precision and recall values calculated for each class shown in Table 12 imply that the model with the correcting vector and with the original ratio should be applied, since it gave higher values both for precision and recall for all classes. It had the lowest values of false negatives for classes 0 and 2, which means it works best in recognizing those classes. The recall for class 1 was slightly worse, compared to the results for the default outcome; but, in the case of that class, we were highly concerned about false positives, as we did not want to badly classify images from classes 0 and 2. We can confirm the proposal by calculating the F1 micro- and macro-scores.

The F1 micro- and macro-scores presented in Table 13 indicate that our assumption was correct, and the correcting vector with original training ratio should be applied. Even though the effect of applying the correcting vector to Custom Vision model was not significant, since the number of observations in classes 0 and 2 might have been too small to teach the model to recognize that classes and its certainty that observations belong to class 1 is too high, the results from that the test dataset would be adjusted by it and compared with the CNN model selected in the next section.

### 3.5. CNN Model

#### 3.5.1. Structure

We proposed a CNN model with the application of a convolution block consisting of two subsequent convolutional layers with a kernel size of (3×3) and stride =1, detected by the rectified linear unit activation function and summarized by a (2×2) max pooling layer. The exact details of the model structure are shown in Table 14.

So, we have four sections: (Conv2D, Conv2D, and MaxPooling), the flattened layer, two shrinking dense layers, and a final classification dense layer. We tested models having four different structures:

- Basic model: [Conv2D, Conv2D, and MaxPooling] ×1, flattened, dense ×3
- Model layers2: [Conv2D, Conv2D, and MaxPooling] ×2, flattened, dense ×3
- Model layers3: [Conv2D, Conv2D, and MaxPooling] ×3, flattened, dense ×3
- Model layers4: [Conv2D, Conv2D, and MaxPooling] ×4, flattened, dense × 3

So, in each model, we added one (Conv2D, Conv2D, and MaxPooling) section. For each of these models, we tested different batch sizes from the range (8, 16, 32, 64, 128, 256, 512) and trained it for 12 epochs. Then, we chose the six best models, based on two criteria: the categorical cross-entropy loss and the highest minimum AUC value, described in Section 2.5. Based on both scores, we chose the six best models and generated more-detailed results to determine the best one.

#### 3.5.2. Optimization

**Figure 9:**
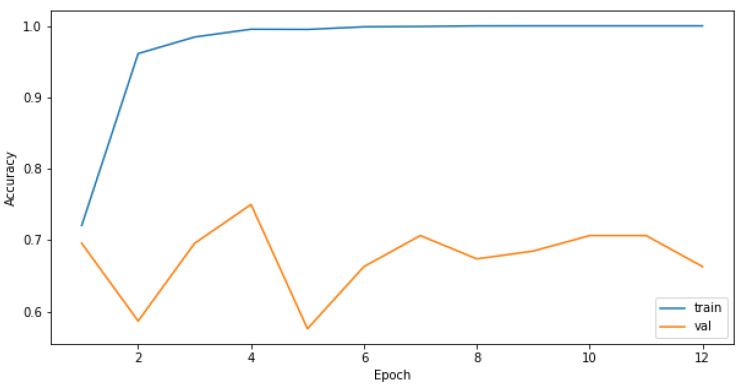
Model accuracy for the model with 1 layer and a batch size of 256 in relation to different number of epochs used to train the CNN.

**Figure 10:**
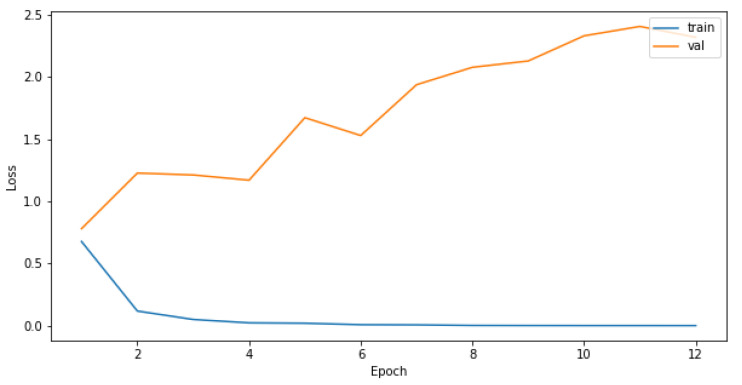
Model loss for the model with 1 layer and a batch size of 256 in relation to different number of epochs used to train the CNN.

We observed validation loss during training of each model described in Section 3.5.1. Then, we chose hyperparameters’ configurations giving the lowest loss on the validation set. The validation-loss criterion achieved the best results when the batch size of data applied in each training step was large, albeit with a small number of epochs, as seen in Figure 9 and Figure 10. The model performance measured as a log loss gained no improvement after adding more epochs for the training set, and it was worse in the case of the validation sets. Even though the convergence did not appear, models trained on a small number of epochs can give satisfying results.

The top three models had only one convolution layer, which concluded that the models with larger structures were overfitting with more epochs of training. One reason for the better performance of shallow networks, when compared to deep networks, could be the fact that the input data were created in a reproducible environment. All the photographs were centred and appropriately cropped, which allowed shallow networks to base their prediction on simple patterns, and the process did not need the advantages that deeper structures of neural networks provide. The results for the 10 best models are presented in Table 15.

Similarly, we calculated the minimum AUC for each model and the results for the ten best solutions, based on this criterion; they are presented in Table 16. The minimal AUC criterion revealed an affinity for models with smaller batch sizes, where nowhere in the top scoring models could a model with only one convolution layer be found. The number of training epochs did not seem to matter for this criterion, as both smaller and larger values appeared within the top-scoring solutions.

Based on this information, we chose six models, which are listed in Table 17. Regarding further analysis of other means of model evaluation, we checked which of the aforementioned criteria produced better results when taking into consideration the overall performance of the model on the testing data.

For the best six models listed above, we tested them by also removing the first dense layer. The results in Table 18 show that we achieved better scores without this layer for two models: 3 and 6. We chose the best option of each model for further analysis.

**Figure 11:**
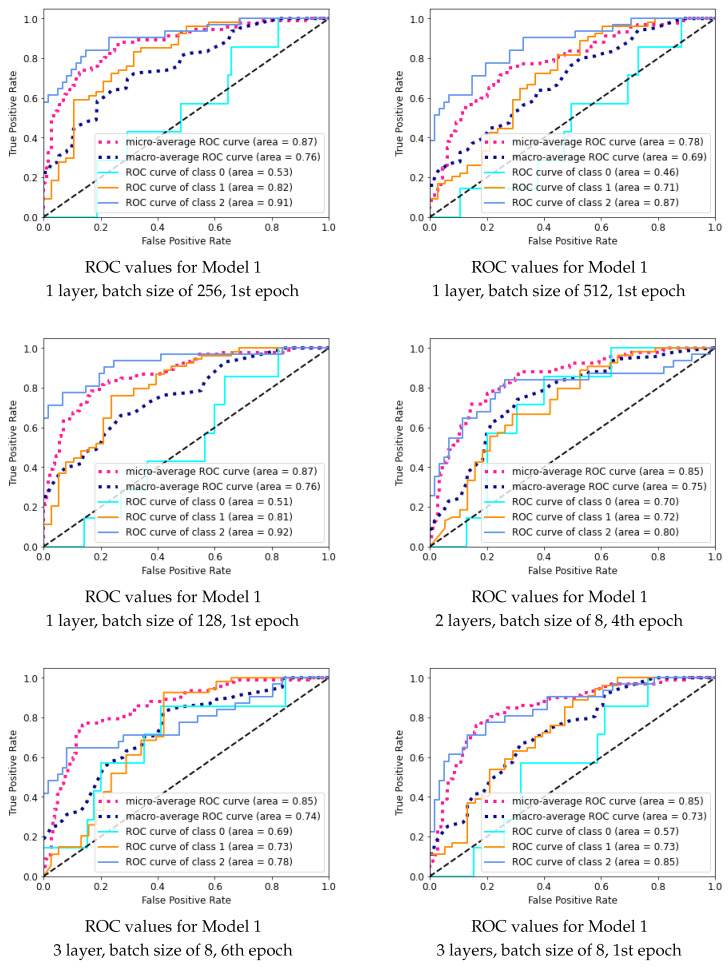
ROC curves for the 6 best models.

We present further results for the chosen six of the best models. Figure 11 gathers the ROC curves based on the results generated by all models on the validation dataset.

As described in Section 2.5, five AUC models allowed us to assess the quality of the models, based on the characteristic for every single class with two supplemental metrics, which allowed us to summarize the performance of every model on the whole dataset.

The AUC scores for class 0 were usually low, except for models 4 and 5. For the other models, the characteristic for this class could even be described as a random model when taking the finest drilled holes into consideration. This was unwanted, but inevitable, as the validation dataset consisted only of seven observations, which may be very similar to the examples provided in class 1. The scores achieved for this class were notably higher for models 4–6, which were chosen based on their minimum AUC values.

The scores of classes 1 and 2 were solid in all models, with higher scores almost always achieved in models 1–3, which were chosen based on the loss criteria. Good model recognition between classes 1 and 2 indicated that the data generated by the GANs were not misleading.

The performance of one model (model 2) could already be discarded from this analysis, since its micro- and macro-averages were substantially lower than for the other models, which means that, on average, the predictions generated from this model were of lower quality.

Based on the confusion matrices shown in Table 19, we can see that, for all models, class 1 was well represented, while class 2 was best predicted for the first, the third, and the fifth model. Moreover, only the fifth model was able to recognize correctly class 0 and seemed to be the best in terms of this criterion.

The precision and recall per class for all models are shown in Table 20. The numbers again suggest that only model 5 could predict class 0. Models 1, 3, and 5 had the best statistics of recall for class 2 (a small number of false negatives) and did not overpredict class 1 (a small number of false positives). The F1 micro- and macro-scores should confirm our assumptions.

In case of the F1 micro- and macro-scores presented in Table 21, which show the ability to differentiate the less-represented class, the highest values were noticed for models 1, 3, and 5. The F1 macro-score was very low for each model, which was not surprising, since in confusion matrices almost none of the models were able to classify class 0 correctly. In the next subsection, we focus on applying the correcting vector to this model to boost the results for less-represented classes.

#### 3.5.3. Further Results for the Best Models with Correcting Vector

In the previous section, we saw that, although precision and recall were fairly high for classes 1 and 2, the results for class 0 were not satisfying. Judging by the AUC score and the ROC curve, it may be possible to find a better metgod to map the probabilities returned by the model to classes.

After multiplying each vector of probabilities $p_{i}$ by the correcting vector π created in two ways (with the original training ratio and with the multiplied training ratio), we obtained the confusion matrices for the selected models shown in Table 22 and Table 23. We can see that the classification of classes 0 and 2 was better now for all the models except model 5 and that the classification for class 1 was always worse. Applying the correcting vector based on the multiplied training ratio makes no sense for any model. It seems that there is no correction needed for model 5 and that the correction with the original ratios would best improve the result for models 1 and 3. To confirm our assumptions and to select the best final model, we compared the precision, the recall, and the F1 micro- and macro-scores for each model.

The comparison of precision and recall presented in Table 24 suggests that if we want to choose the model that has the minimal false-positive ratio for class 0, we should choose model 5, but in our case we believe that it is more important to have the minimum number of false negatives predicted. If we take into account the statistics for class 2, our aim was again to reach the highest possible value for recall, so model 1 with the correcting vector of the original ratio performed the best. In the case of class 1 we wanted to minimize the value of false positives and maximize the precision, so the numbers suggest that models 1 and 3 worked the finest. Summing up, it seems that model 1 best accomplished all of our requirements.

In Table 25, the comparison of the F1 micro- and macro-scores for each model is presented. The statistics here were relatively low, which comes from the fact that the measures take into account both the precision and the recall for all the classes, while in our case we wanted to maximize the recall for classes 0 and 2 and the precision for class 1. Therefore, we are not bound by the results of this measure and selected model 1 for the comparison with the Microsoft Custom Vision tool.

## 4. Results

In this section, we compare our model chosen as the best one, that is, model 1, against the Microsoft Custom Vision model with the correction vectors classifying observations on the independent test set.

### Comparing the Custom Vision Solution and the CNN Model

The first step is the comparison of confusion matrices presented in Table 26. Classes 0 and 2 were considerably better predicted by the CNN model built from scratch. If we take into account class 1, it is definitely better predicted by the Custom Vision tool. In the next step, we look at the precision and recall for each class.

Based on infromation from Table 27, class 0 had higher values for precision in the Custom Vision model, but recall was higher in the CNN model, which means that the model built from scratch more often assigned observations to class 0. In the CNN model, the observations from class 1 were often mistaken with class 0. The same pattern can be seen for class 2; the CNN model had a higher true-positive rate. If we want to maximize recall for classes 0 and 2 and precision for class 1, the CNN Model should be chosen.

In the Table 28, we can see that the Custom Vision model was better than CNN model built from scratch in terms of the averaged F1 micro- and macro- scores. The value was higher, because the Azure model was much more accurate in predicting class 1, which was the most numerous.

Considering all of the statistics and analyses that were made, it is hard to unambiguously assess which solution is the best. It highly depends on the business condition that should be fulfilled by the model. If we want to focus on classifying the best and the worst class, the proposed CNN model is considerably superior. Correct assignment of theses classes seem to be more important in a given task. What is more, the designed model is relatively simple and easy to build and train, and it gives satisfying results with a relatively small amount of data. On the other hand, the Azure model works better with the most numerous class and requires no statistical knowledge to be built. As we do not have access to the documentation of the Azure solution, it is hard to say how many images were provided to pretrain the tool, but we can assume with a high degree of certainty that it was much more than the 15,689 observation given to the CNN model.

## 5. Conclusions

The aim of this study was to adopt a simple convolution neural network to a multiclassification problem and to compare it with the model provided by the Microsoft Azure platform (Microsoft Custom Vision). What is new is that we exploited many measures based on the confusion matrix instead of comparing the accuracies between models. Moreover, a correcting vector was applied and evidenced to be legitimate to adjust the predictions of less-represented classes.

In order to train the model, some data-augmentation techniques were applied. First of all, we developed the method of combining features from existing images, by taking the mathematical average of corresponding points in numerical matrices of two images. It was mostly useful in the case of the least-represented classes, which could not by multiplied sufficiently with data-augmentation transformations proposed in the study. What is more, it was proved that the general adversarial networks worked well for multiplication of such data like the drill images. The generated data turned out to be of good quality and were sufficiently accurate to be used to train the model, which was able to correctly predict the quality of the drill from an independent sample, which was evinced both by application of the Azure Custom Vision model and the CNN model built from scratch.

Moreover, in case of such uncomplicated images like drill holes, the simple CNN model with only one layer was sufficient to correctly solve the multiclassification problem. More sophisticated solutions, like CNN models with multiple layers or Microsoft Custom Vision tool gave no or small improvements in model performance, while it is highly more complicated to understand their mechanisms and structures.

The results could be improved if the provided dataset of real images was more substantial. This approach was originally rejected, in favour of multiplying smaller datasets with data augmentation, due to the high amount of manual cost and the tedious work that is required to generate photographs with corresponding labels.

One way to discard this high business cost potential would be to use an automated method of generating labels on unknown real-life samples. The concept of semi-supervised learning assumes that fully labelled data existing for only fraction of the database (like 0.08% of examples, as described in [39]) gets passed as an input to a neural network that, after training, is able to generate mostly accurate labels on the unknown piece of gathered data. Labels generated in this way are then used as an input for further process of training other models. The results from this method are promising [40,41] and are left as an avenue for further research.

There is still a significant amount of work to be done on the analysed dataset. The model can be trained on a fully balanced dataset, which can improve the result for classes 0 and 2, or with the use of semi-supervised learning and other data augmentation techniques, like applying additional transformations that would extract specific regions of interest from the provided images. What is more, the legitimacy of classifying images into three classes should be tested, both from the business and the statistical point of view. Binary classification is much simpler and can give better results, especially when the number of images in class 0 is dramatically low.

## Figures and Tables

**Table 1:** Sizes of the classes in the dataset.

Class	Description	Number of Objects
Class 0	very fine	33
Class 1	acceptable	271
Class 2	unacceptable	155
**Total**		459

**Table 2:** Number of observations in the dataset and its distribution between classes.

Class	Train	Validation	Test
Class 0	19	7	7
Class 1	162	54	55
Class 2	93	31	31
**Sum**	274	92	93

**Table 3:** Confusion matrix for CNN Model on validation set trained on original dataset (274 observations).

True/Predicted	Class 0	Class 1	Class 2
Class 0	0/7	7/7	0/7
Class 1	0/54	54/54	0/54
Class 2	0/31	31/31	0/31

**Table 4:** Number of observations in the training dataset after the combination of images was applied to original data.

Class	Train
Class 0	361
Class 1	1597
Class 2	521
**Sum**	2479

**Table 5:** Number of observations in the train dataset and its distribution between classes after data augmentation.

Class	Train
Class 0	1440
Class 1	3190
Class 2	2080
**Sum**	6710

**Table 6:** Structure of GAN generator model.

Layer	Output Shape	Parameters
Dense	(None, 3200)	259,200
LeakyReLU	(None, 3200)	0
Reshape	(None, 5, 5, 128)	0
Conv2DTranspose	(None, 10, 10, 128)	262,272
LeakyReLU	(None, 10, 10, 128)	0
Conv2DTranspose	(None, 20, 20, 128)	262,272
LeakyReLU	(None, 20, 20, 128)	0
Conv2DTranspose	(None, 40, 40, 128)	262,272
LeakyReLU	(None, 40, 40, 128)	0
Conv2DTranspose	(None, 80, 80, 128)	262,272
LeakyReLU	(None, 80, 80, 128)	0
Conv2D	(None, 80, 80, 1)	1153
**Total params**	1,309,441	
**Trainable params**	1,309,441	

**Table 7:** Structure of the GAN discriminator model.

Layer	Output shape	Parameters
Conv2D	(None, 40, 40, 64)	640
LeakyReLU	(None, 40, 40, 64)	0
Dropuot	(None, 40, 40, 64)	0
Conv2D	(None, 20, 20, 64)	36,928
LeakyReLU	(None, 20, 20, 64)	0
Dropuot	(None, 20, 20, 64)	0
Flatten	(None, 25, 600)	0
Dense	(None, 1)	26,601
**Total params**	63,169	
**Trainable params**	0	
**Non-trainable params**	63,169	

**Table 8:** Structure of the GAN model.

Layer	Output Shape	Parameters
Generator model	(None, 80, 80, 1)	1,309,441
Discriminator model	(None, 1)	63,169
**Total params**	1,372,610	
**Trainable params**	1,309,441	
**Non-trainable params**	63,169	

**Table 9:** Number of classes in the training dataset generated by the GANs.

Class	Train
Class 0	1400
Class 1	3100
Class 2	2000
**Sum**	6500

**Table 10:** Number of observations in the dataset and its distribution between classes.

Class	Train	Validation	Test
Class 0	3201	7	7
Class 1	7887	54	55
Class 2	4601	31	31
**Sum**	15,689	92	93

**Table 11:** Confusion matrices for the Custom Vision model on the validation set with different correcting vectors.

Custom Vision Model, Default Results			
**True/Predicted**	**Class 0**	**Class 1**	**Class 2**
Class 0	1/7	6/7	0/7
Class 1	0/54	54/54	0/54
Class 2	0/31	19/31	12/31
**Custom Vision Model, Original Training Vector**			
**True/Predicted**	**Class 0**	**Class 1**	**Class 2**
Class 0	3/7	4/7	0/7
Class 1	2/54	52/54	0/54
Class 2	0/31	16/31	15/31
**Custom Vision Model, Multiplied Training Vector**			
**True/Predicted**	**Class 0**	**Class 1**	**Class 2**
Class 0	2/7	5/7	0/7
Class 1	2/54	52/54	0/54
Class 2	0/31	16/31	15/31

**Table 12:** Precision and recall per class for the Microsoft Custom Vision model on the validation set with different correction vectors.

Microsoft Custom Vision	Class 0	Class 1	Class 2
Precision (default)	1.00	0.68	1.00
Recall (default)	0.14	1.00	0.39
Precision (original ratio)	0.60	0.72	1.00
Recall (original ratio)	0.43	0.96	0.48
Precision (training ratio)	0.50	0.71	1.00
Recall (training ratio)	0.29	0.96	0.48

**Table 13:** F1 scores for the Custom Vision model on the validation set with different correcting vectors.

Model	F1 Micro	F1 Macro
Model with default results	0.73	0.54
Model with original training vector	0.76	0.66
Model with multiplied training vector	0.75	0.61

**Table 14:** Structure of the proposed CNN model; example of a model with 4 layers.

Layer	Output Shape	Parameters
Conv2D	(None, 78, 78, 32)	320
Conv2D	(None, 76, 76, 32)	9248
MaxPooling	(None, 38, 38, 32)	0
Conv2D	(None, 36, 36, 64)	18,496
Conv2D	(None, 34, 34, 64)	36,928
MaxPooling	(None, 17, 17, 64)	0
Conv2D	(None, 15, 15, 128)	73,856
Conv2D	(None, 13, 13, 128)	147,584
MaxPooling	(None, 6, 6, 128)	0
Conv2D	(None, 4, 4, 128)	295,168
Conv2D	(None, 2, 2, 128)	590,080
MaxPooling	(None, 1, 1, 128)	0
Flatten	(None, 256)	0
Dense	(None, 128)	32,896
Dense	(None, 64)	8256
Dense	(None, 3)	195
**Total params**	1,213,027	
**Trainable params**	1,213,027	
**Non-trainable params**	0	

**Table 15:** Parameter configuration and model loss for the training and validation sets.

Number	Batch	Epochs	Train	Validation
of Layers	Size		Loss	Loss
1	256	1	0.51	0.72
1	512	1	0.86	0.89
1	128	1	0.33	0.90
4	512	3	0.44	0.92
3	512	2	0.59	0.93
4	16	4	1.03	0.93
2	512	1	0.88	0.93
4	128	2	0.29	0.97
1	512	5	0.05	0.98
3	512	4	0.23	0.98

**Table 16:** Parameter configuration, AUC for each class, and minimum AUC for the validation set.

Number of	Batch	Epochs	AUC	AUC	AUC	min
Layers	Size		Class 0	Class 1	Class 2	AUC
2	8	4	0.70	0.72	0.80	0.70
3	8	6	0.69	0.73	0.78	0.69
3	8	1	0.73	0.68	0.78	0.68
4	8	11	0.68	0.71	0.77	0.68
3	8	11	0.67	0.71	0.76	0.67
3	16	8	0.67	0.68	0.79	0.67
3	8	5	0.67	0.66	0.79	0.66
4	8	10	0.69	0.66	0.75	0.66
2	8	6	0.66	0.68	0.77	0.66
3	16	3	0.66	0.75	0.80	0.66

**Table 17:** Description of chosen models based on the validation loss and the highest minimal AUC value.

Model	Number	Batch	Epochs	Validation	min
	of Layers	Size		Loss	AUC
1	1	256	1	0.72	0.53
2	1	512	1	0.89	0.46
3	1	128	1	0.90	0.55
4	2	8	4	2.61	0.70
5	3	8	6	2.11	0.69
6	3	8	1	1.76	0.68

**Table 18:** Validation loss for the 6 best models after removing the third dense layer.

Model	Number	Batch	Epochs	3rd Dense	Validation
	of Layers	Size		Layer Present	Loss
1	1	256	1	Yes	0.72
1	1	256	1	No	1.05
2	1	512	1	Yes	0.89
2	1	512	1	No	1.00
3	1	128	1	Yes	0.90
3	1	128	1	No	0.75
4	2	8	4	Yes	2.61
4	2	8	4	No	3.27
5	3	8	6	Yes	2.11
5	3	8	6	No	2.89
6	3	8	1	Yes	1.76
6	3	8	1	No	1.55

**Table 19:** Confusion matrices for models 1–6.

Model 1			
**True/Predicted**	**Class 0**	**Class 1**	**Class 2**
Class 0	0/7	5/7	2/7
Class 1	5/54	46/54	3/54
Class 2	0/31	10/31	21/31
**Model 2**			
**True/Predicted**	**Class 0**	**Class 1**	**Class 2**
Class 0	0/7	7/7	0/7
Class 1	0/54	54/54	0/54
Class 2	0/31	29/31	2/31
**Model 3**			
**True/Predicted**	**Class 0**	**Class 1**	**Class 2**
Class 0	0/7	7/7	0/7
Class 1	8/54	46/54	0/54
Class 2	2/31	8/31	21/31
**Model 4**			
**True/Predicted**	**Class 0**	**Class 1**	**Class 2**
Class 0	0/7	7/7	0/7
Class 1	1/54	49/54	4/54
Class 2	0/31	14/31	17/31
**Model 5**			
**True/Predicted**	**Class 0**	**Class 1**	**Class 2**
Class 0	1/7	5/7	1/7
Class 1	1/54	47/54	6/54
Class 2	0/31	11/31	20/31
**Model 6**			
**True/Predicted**	**Class 0**	**Class 1**	**Class 2**
Class 0	0/7	6/7	1/7
Class 1	2/54	51/54	1/54
Class 2	0/31	16/31	15/31

**Table 20:** Precisions and recalls for models 1–6.

Model 1	Class 0	Class 1	Class 2
Precision	0.00	0.75	0.81
Recall	0.00	0.85	0.68
**Model 2**			
Precision	0.00	0.60	1.00
Recall	0.00	1.00	0.06
**Model 3**			
Precision	0.00	0.75	1.00
Recall	0.00	0.85	0.68
**Model 4**			
Precision	0.00	0.70	0.81
Recall	0.00	0.91	0.55
**Model 5**			
Precision	0.50	0.75	0.74
Recall	0.14	0.87	0.65
**Model 6**			
Precision	0.00	0.70	0.88
Recall	0.00	0.94	0.48

**Table 21:** F1 scores for the 6 best models.

Model	F1 Micro	F1 Macro
Model 1	0.73	0.51
Model 2	0.61	0.29
Model 3	0.73	0.54
Model 4	0.72	0.48
Model 5	0.74	0.57
Model 6	0.72	0.48

**Table 22:** Confusion matrices for models 1, 3, and 5, corrected with the original training vector on the validation set.

Confusion Matrix, Model 1, Original Training Vector			
**True/Predicted**	**Class 0**	**Class 1**	**Class 2**
Class 0	4/7	1/7	2/7
Class 1	37/54	12/54	5/54
Class 2	5/31	3/31	23/31
**Confusion Matrix, Model 3, Original Training Vector**			
**True/Predicted**	**Class 0**	**Class 1**	**Class 2**
Class 0	3/7	2/7	2/7
Class 1	37/54	15/54	2/54
Class 2	7/31	2/31	22/31
**Confusion Matrix, Model 5, Original Training Vector**			
**True/Predicted**	**Class 0**	**Class 1**	**Class 2**
Class 0	1/7	5/7	1/7
Class 1	3/54	43/54	8/54
Class 2	0/31	11/31	20/31

**Table 23:** Confusion matrices for models 1, 3, and 5, corrected with multiplied training vector on the validation set.

Confusion Matrix, Model 1, Multiplied Training Vector			
**True/Predicted**	**Class 0**	**Class 1**	**Class 2**
Class 0	1/7	4/7	2/7
Class 1	14/54	35/54	5/54
Class 2	2/31	5/31	24/31
**Confusion Matrix, Model 3, Multiplied Training Vector**			
**True/Predicted**	**Class 0**	**Class 1**	**Class 2**
Class 0	1/7	4/7	2/7
Class 1	16/54	36/54	2/54
Class 2	2/31	6/31	23/31
**Confusion Matrix, Model 5, Multiplied Training Vector**			
**True/Predicted**	**Class 0**	**Class 1**	**Class 2**
Class 0	1/7	5/7	1/7
Class 1	1/54	45/54	8/54
Class 2	0/31	11/31	20/31

**Table 24:** Precisions and recalls for models 1, 3, and 5 with correcting vectors applied.

Model 1	Class 0	Class 1	Class 2
Precision (default)	0.00	0.75	0.81
Recall (default)	0.00	0.85	0.68
Precision (original ratio)	0.09	0.75	0.77
Recall (original ratio)	0.57	0.22	0.74
Precision (training ratio)	0.06	0.80	0.77
Recall (training ratio)	0.14	0.65	0.77
**Model 3**	**Class 0**	**Class 1**	**Class 2**
Precision (default)	0.00	0.75	1.00
Recall (default)	0.00	0.85	0.68
Precision (original ratio)	0.06	0.79	0.85
Recall (original ratio)	0.43	0.28	0.71
Precision (training ratio)	0.05	0.78	0.85
Recall (training ratio)	0.14	0.67	0.74
**Model 5**	**Class 0**	**Class 1**	**Class 2**
Precision (default)	0.50	0.75	0.74
Recall (default)	0.14	0.87	0.65
Precision (original ratio)	0.25	0.73	0.69
Recall (original ratio)	0.14	0.80	0.65
Precision (training ratio)	0.50	0.74	0.69
Recall (training ratio)	0.14	0.83	0.65

**Table 25:** F1 micro- and macro-scores for the best models with different correcting vectors.

Model	F1 Micro	F1 Macro
Model 1 (default)	0.73	0.51
Model 1 (original ratio)	0.42	0.42
Model 1 (training ratio)	0.65	0.52
Model 3 (default)	0.73	0.54
Model 3 (original ratio)	0.43	0.43
Model 3 (training ratio)	0.65	0.53
Model 5 (default)	0.74	0.57
Model 5 (original ratio)	0.70	0.54
Model 5 (training ratio)	0.72	0.56

**Table 26:** Confusion matrices for best CNN Model and Custom Vision model corrected with original training vector on test set.

Confusion Matrix, Best Cnn Model, Original Training Vector			
**True/Predicted**	**Class 0**	**Class 1**	**Class 2**
Class 0	6/7	1/7	0/7
Class 1	19/55	26/55	10/55
Class 2	1/31	2/31	28/31
**Confusion Matrix, Custom Vision Model, Original Training Vector**			
**True/Predicted**	**Class 0**	**Class 1**	**Class 2**
Class 0	4/7	3/7	0/7
Class 1	1/55	52/55	2/55
Class 2	0/31	11/31	20/31

**Table 27:** Precisions and recalls for best CNN Model and Custom Vision model corrected with original training vector on the test set.

CNN Model	Class 0	Class 1	Class 2
Precision	0.23	0.89	0.74
Recall	0.86	0.47	0.90
**Custom Vision Model**			
Precision	0.80	0.79	0.91
Recall	0.57	0.95	0.65

**Table 28:** F1 micro- and macro-scores for the best CNN Model and the Custom Vision model.

Model	F1 Micro	F1 Macro
CNN Model	0.65	0.60
Custom Vision Model	0.82	0.76

## References

[1] Kurek J., Kruk M., Osowski S., Hoser P., Wieczorek G., Jegorowa A., Górski J., Wilkowski J., Śmietańska K., Kossakowska J. (2016). Developing automatic recognition system of drill wear in standardlaminated chipboard drilling process. Bull. Pol. Acad. Sci..

[2] Jemielniak K., Urbański T., Kossakowska J., Bombiński S. (2012). Tool condition monitoring based on numerous signal feature. Int. J. Adv. Manuf. Technol..

[3] Panda S.S., Singh A.K., Chakraborty D., Pal S.K. (2006). Drill wear monitoring using back propagationneural network. J. Mater. Process. Technol..

[4] Kuo R.J. (2000). Multi-sensor integration for on-line tool wear estimation through artificial neural net-works and fuzzy neural network. Eng. Appl. Artif. Intell..

[5] Kurek J., Wieczorek G., Świderski B., Kruk M., Jegorowa A., Osowski S. Transfer learning in recognition of drill wear using convolutional neural network. Proceedings of the 18th International Conference on Computational Problems of Electrical Engineering (CPEE).

[6] Kurek J., Antoniuk I., Górski J., Jegorowa A., Świderski B., Kruk M., Wieczorek G., Pach J., Orłowski A., Aleksiejuk-Gawron J. (2019). Data Augmentation Techniques for Transfer Learning Improvement in Drill Wear Classification Using Convolutional Neural Network. Mach. Graph. Vis..

[7] Kurek J., Antoniuk I., Świderski B., Jegorowa A., Bukowski M. (2019). Application of Siamese Networks to the Recognition of the Drill Wear State Based on Images of Drilled Holes. Sensors.

[8] Kurek J., Świderski B., Jegorowa A., Kruk M., Osowski S. Deep learning in assessment of drill condition on the basis of images of drilled holes. Proceedings of the SPIE 10225 Eighth International Conference on Graphic and Image Processing (ICGIP 2016).

[9] Bowles C., Chen L., Guerrero R., Bentley P., Gunn R., Hammers A., Dickie D., Hernández M., Wardlaw J., Rueckert D. (2018). GAN Augmentation: Augmenting Training Data using Generative Adversarial Networks. arXiv.

[10] Salimans T., Goodfellow I., Zaremba W., Cheung V., Radford A., Chen X., Chen X., Lee D., Sugiyama M., Luxburg U., Guyon I., Garnett R. (2016). Improved Techniques for Training GANs. Advances in Neural Information Processing Systems.

[11] Hossein H.S., Mohammad R., Mohsen F., Mohammad S. (2018). Lets Keep It Simple: Using Simple Architectures to Outperform Deeper and More Complex Architectures. arXiv.

[12] Ba J., Caruana R., Ghahramani Z., Welling M., Cortes C., Lawrence N., Weinberger K.Q. (2014). Do Deep Nets Really Need to be Deep?. Advances in Neural Information Processing Systems.

[13] Hinton G., Vinyals O., Dean J. Distilling the Knowledge in a Neural Network. Proceedings of the NIPS Deep Learning and Representation Learning Workshop.

[14] Wu F., Souza A., Zhang T., Fifty C., Yu T., Weinberger K. Simplifying Graph Convolutional Networks. Proceedings of the 36th International Conference on Machine Learning.

[15] Jegorowa A., Kurek J., Antoniuk I., Dołowa W., Bukowski M., Czerniak P. (2021). Deep learning methods for drill wear classification based on images of holes drilled in melamine faced chipboard. Wood Sci. Technol..

[16] Mahani A., Ali A.R.B. (2019). Classification problem in imbalanced datasets. Recent Trends Computational Intelligence.

[17] Wang L., Han M., Li X., Zhang N., Cheng H. (2021). Review of Classification Methods on Unbalanced Data Sets. IEEE Access.

[18] Pejčinović M. A Review of Custom Vision Service for Facilitating an Image Classification. Proceedings of the Central European Conference on Information and Intelligent Systems, Faculty of Organization and Informatics.

[19] Taylor L., Nitschke G. (2017). Improving Deep Learning using Generic Data Augmentation. arXiv.

[20] Gu S., Pednekar M., Slater R. (2019). Improve Image Classification Using Data Augmentation and Neural Networks. SMU Data Science Review 2.2.

[21] Bui H., Lech M., Cheng E., Neville K., Burnett I. Using Grayscale Images for Object Recognition with Convolutional-Recursive Neural Network. Proceedings of the IEEE Sixth International Conference on Communications and Electronics (ICCE).

[22] Perez L., Wang J. (2017). The effectiveness of data augmentation in image classification using deep learning. arXiv.

[23] Shorten C., Khoshgoftaar T.M. (2019). A survey on image data augmentation for deep learning. J. Big Data.

[24] Goodfellow I., Pouget-Abadie J., Mirza M., Xu B., Warde-Farley D., Ozair S., Courville A., Bengio Y., Ghahramani Z., Welling M., Cortes C., Lawrence N., Weinberger K.Q. (2014). Generative Adversarial Nets. Advances in Neural Information Processing Systems.

[25] Durall R., Chatzimichailidis A., Labus P., Keuper J. (2020). Combating Mode Collapse in GAN training: An Empirical Analysis using Hessian Eigenvalues. arXiv.

[26] Mirza M., Osindero S. (2014). Conditional generative adversarial nets. arXiv.

[27] Shu R., Bui H., Ermon S. AC-GAN Learns a Biased Distribution. Proceedings of the NIPS Workshop on Bayesian Deep Learning.

[28] Zhou P., Xie L., Ni B., Geng C., Tian Q. Omni-GAN: On the Secrets of cGANs and Beyond. Proceedings of the IEEE/CVF International Conference on Computer Vision.

[29] Simard P.Y., Steinkraus D., Platt J.C. Best practices for convolutional neural networks applied to visual document analysis. In Proceeedings of the International Conference on Doc- ument Analysis and Recogntion (ICDAR).

[30] Mikołajczyk A., Grochowski M. Data augmentation for improving deep learning in image classification problem. Proceedings of the 2018 International Interdisciplinary PhD Workshop (IIPhDW).

[31] Thoma M. (2017). Analysis and optimization of convolutional neural network architectures. arXiv.

[32] Simonyan K., Zisserman A. (2014). Very deep convolutional networks for large-scale image recognition. arXiv.

[33] Goodfellow I., Bengio Y., Courville A., Bengio Y. (2016). Deep Learning.

[34] Ali O., Ishak M.K. (2020). Bringing intelligence to IoT Edge: Machine Learning based Smart City Image Classification using Microsoft Azure IoT and Custom Vision. J. Phys. Conf. Ser..

[35] Documentation Page for Keras Deep Learning Library for Python. https://keras.io.

[36] Documentation Page for Scikit-Learn Package Regarding Receiver Operating Curve. https://scikit-learn.org/stable/auto_examples/model_selection/plot_roc.html.

[37] Buda M., Maki A., Mazurowski M.A. (2018). A systematic study of the class imbalance problem in convolutional neural networks. Neural Netw..

[38] Richard M.D., Lippmann R.P. (1991). Neural network classifiers estimate Bayesian a posteriori probabilities. Neural Comput..

[39] Protopapadakis E., Doulamis A., Doulamis N., Maltezos E. (2021). Stacked autoencoders driven by semi-supervised learning for building extraction from near infrared remote sensing imagery. Remote Sens..

[40] Tarvainen A., Valpola H. (2017). Mean teachers are better role models: Weight-averaged consistency targets improve semi-supervised deep learning results. arXiv.

[41] Baur C., Albarqouni S., Navab N. Semi-supervised deep learning for fully convolutional networks. Proceedings of the International Conference on Medical Image Computing and Computer-Assisted Intervention.

